# Fast Implementation of Approximated Maximum Likelihood Parameter Estimation for Frequency Agile Radar under Jamming Environment

**DOI:** 10.3390/s20072022

**Published:** 2020-04-03

**Authors:** Yang Zhao, Jianxin Wu, Zhiyong Suo, Xiaoyu Liu

**Affiliations:** 1 National Laboratory of Radar Signal Processing, Xidian University, Xidian 710071, China; yzhao_97@stu.xidian.edu.cn (Y.Z.); LiuXY1513@126.com (X.L.); 2 School of Electronics and Communication Engineering, Sun Yat-sen University, Guangzhou 510275, China; wujx65@mail.sysu.edu.cn

**Keywords:** maximum likelihood estimation, frequency agility, jamming suppression

## Abstract

A computationally efficient target parameter estimation algorithm for frequency agile radar (FAR) under jamming environment is developed. First, the barrage noise jamming and the deceptive jamming are suppressed by using adaptive beamforming and frequency agility. Second, the analytical solution of the parameter estimation is obtained by a low-order approximation to the multi-dimensional maximum likelihood (ML) function. Due to that, fine grid-search (FGS) is avoided and the computational complexity is greatly reduced.

## 1. Introduction

With the development of new countermeasure technology, radar is faced with an increasingly complex electromagnetic environment [1]. Therefore, how to eliminate the negative influences using the complex environment is the precondition of successful target identification [2]. It is well known that the frequency agile radar (FAR) that adopts the pulse-to-pulse random frequency agility over shorter intervals can improve the performance in target detection [3,4], and its thumbtack ambiguity function [5] implies that one is able to obtain high range–velocity resolution and avoid range–velocity coupling problems which are present in traditional frequency agility waveforms [6]. Thus, the FAR is an important anti-electronic countermeasure [7,8].

As for the target detection, the early studies were mainly focused on incoherent integration such as the Hough transform [9] and the Radon transform based methods [10]. However, it has integration loss compared with the coherent method. Consequently, some researchers proposed the target detection method based on coherent integration [11]. In [12,13], Zhou et al. proposed an adaptive monopulse (AM) algorithm based on combining sum–difference and auxiliary beam at subarray level for anti-jamming. In [14], based on a wideband model, Qian et al. proposed a wideband scale-based Radon–Fourier transform (WSRFT) method for coherent integration of high-speed targets, but only for fixed carrier frequency radars. In [7], Wang et al. proposed an improved frequency agile coherent radon transform (FA-CRT) scheme to cope with the long-time coherent integration in frequency agile radar. In [15], P. Huang et al. proposed a method for removing the residual coupling between the agile carrier frequency and slow-time by using Keystone Transform (KT) between different bursts to achieve coherent accumulation. In [16], Li et al. proposed a combined range–velocity estimation method based on compressive sensing (CS) in the range–velocity plane, where the CS is employed to reduce the number of channels, therefore, the system complexity and the computational burden are effectively reduced [17].

However, the computational complexities of CRT and CS are very high which greatly limits their applications. In this communication, a computationally efficient algorithm for estimating target parameters of FAR under jamming environment is developed. The barrage noise jamming and the deceptive jamming are firstly suppressed by using adaptive beamforming and frequency agility [18], and then a low-order approximation of the multi-dimensional maximum likelihood (ML) function is employed to estimate target parameters [19]. Due to the fact that the fine grid-search is not necessary anymore, the computational complexity is greatly reduced relative to the conventional grid-search method. The experimental results show that this method has a lower computational complexity at the cost of trivial performance loss.

## 2. System Model Characterization

### 2.1. Signal Model

Assume that there are *M* elements and FAR transmits *N* pulses with random carrier frequencies. The frequency of the nth pulse can be denoted by $f_{n}=f_{0}+k_{n}\Delta f$, where $f_{0}$ is the initial carrier frequency, Δf is the minimal frequency agility interval, and $k_{n}$ controls the rules of how carrier frequency varies. Based on the signal model given in [15], the received signal of the nth pulse at the mth array element after matched filtering can be expressed as

(1) $$s_{mn}\left(t,\tau \right)=a\sin c\left(\Delta f\left(t-\tau_{mn}\right)\right)\exp\left(-j2\pi f_{n}\tau_{mn}\right)$$

where a is the complex amplitude of a moving point-like target, and $\tau_{mn}$ is the target time delay of the nth pulse at the mth array element, which can be written as

(2) $$\tau_{mn}=2\left(r-vnT-\frac{1}{2}md\sin\theta \right)/c$$

where r is the target range, v is the target velocity, d is the array element spacing and θ is the target angle. For simplicity, we define $\eta ={\left[\begin{array}{ccc} r & v & \theta \end{array}\right]}^{T}$ as the target parameter vector. The target data can be assembled in the form of a space–time snapshot

(3) $$\bar{s}\left(\eta \right)=as\left(\eta \right)=a{\left[\begin{array}{cccc} s_{0}^{T} & s_{1}^{T} & \ldots & s_{N-1}^{T} \end{array}\right]}^{T}$$

where $s_{n}={\left[\begin{array}{cccc} \exp\left(-j2\pi f_{n}\tau_{0n}\right) & \exp\left(-j2\pi f_{n}\tau_{1n}\right) & \ldots & \exp\left(-j2\pi f_{n}\tau_{\left(M-1\right)n}\right) \end{array}\right]}^{T}$ is an M×1 spatial steering vector of the nth pulse, the superscript T means the matrix transpose. Meanwhile, the deceptive jamming, which is usually obtained by delaying the transmitting signal for multiple pulse repetition intervals, can be easily suppressed by matched filtering. Thus, the component of deceptive jamming is omitted after matched filtering. Furthermore, the barrage noise jamming often emerges in various radar scenes and should be taken into account here. According to the model given in [20], the jammer space–time snapshot may be written as $x_{J}=\alpha_{J}⊗a_{J}$ and the jammer space–time covariance matrix is $R_{J}=E\left(x_{J}x_{J}^{H}\right)=I_{N}⊗\Phi_{J}$, where $\alpha_{J}$ is a random vector containing the jammer amplitudes, $a_{J}$ is the jamming steering vector, and $\Phi_{J}=\sigma_{J}^{2}a_{J}a_{J}^{H}$ means the jamming spatial covariance matrix, and $\sigma_{J}^{2}$ is the jamming power. In addition, the noise space–time snapshot is denoted by $x_{n}$ and the noise space–time covariance matrix is $R_{n}=E\left[x_{n}x_{n}^{H}\right]=\sigma^{2}I_{MN}$, where $\sigma^{2}$ is the noise power. Finally, the echo data can be expressed as

(4) $$x_{1}=as\left(\eta \right)+x_{0}$$

where $x_{0}=x_{n}+x_{J}$ encompasses the barrage noise jamming and noise, which has the covariance matrix

(5) $$R_{0}=E\left[x_{0}x_{0}^{H}\right]=R_{J}+R_{n}=I_{N}⊗\Phi_{\mathrm{Jn}}$$

where $\Phi_{\mathrm{Jn}}=\Phi_{J}+\sigma^{2}I_{M}$.

### 2.2. ML Parameter Estimation

In this section, a computationally efficient algorithm for target parameters estimation based on the ML method is developed. The joint probability density function of the observed data is given by

(6) $$f\left(x_{1}|\eta \right)=\frac{1}{{\left(2\pi \right)}^{\frac{n}{2}}{\left|R_{0}^{}\right|}^{\frac{1}{2}}}\exp\left(-{\left(x_{1}-as\left(\eta \right)\right)}^{H}R_{0}^{-1}\left(x_{1}-as\left(\eta \right)\right)\right)$$

According to the definition of the likelihood function and ignoring the constant term, the corresponding log-likelihood function is

(7) $$L\left(a,\eta \right)={\left(x_{1}-as\left(\eta \right)\right)}^{H}R_{0}^{-1}\left(x_{1}-as\left(\eta \right)\right)$$

The ML estimate of a can be obtained by setting the derivative of the log-likelihood function with respect to a equal to zero. After some manipulations, we have

(8) $$\hat{a}=\frac{s^{H}\left(\eta \right)R_{0}^{-1}x_{1}}{s^{H}\left(\eta \right)R_{0}^{-1}s\left(\eta \right)}$$

Substituting (8) into (7) yields the ML estimate of η, that is

(9) $$\hat{\eta }=\arg\underset{\eta }{\max}L\left(\eta \right)=\frac{{\left|s^{H}\left(\eta \right)R_{0}^{-1}x_{1}\right|}^{2}}{s^{H}\left(\eta \right)R_{0}^{-1}s\left(\eta \right)}$$

In order to obtain the ML estimate of η, it is necessary to perform a search over the whole parameter grids. However, the three-dimensional fine grid-search brings large computational cost. To reduce the computational complexity, we can resort to a solving equation. As we know, the ML solution should satisfy the following condition: the partial derivatives of the log-likelihood function with respect to these parameters are equal to zero, that is

(10) $$\left[\begin{array}{l} {\dot{L}}_{\theta }\left(\eta \right)\\ {\dot{L}}_{v}\left(\eta \right)\\ {\dot{L}}_{r}\left(\eta \right) \end{array}\right]=\left[\begin{array}{l} 0\\ 0\\ 0 \end{array}\right]$$

where ${\dot{L}}_{\theta }\left(\eta \right)$, ${\dot{L}}_{v}\left(\eta \right)$, ${\dot{L}}_{r}\left(\eta \right)$ represents the first-order partial derivative of L(η) with respect to θ, v and r, respectively. After some manipulations, ${\dot{L}}_{\theta }\left(\eta \right)$ can be written as

(11) $${\dot{L}}_{\theta }\left(\eta \right)=\frac{\left(\Delta \Sigma^{H}+\Delta^{H}\Sigma \right)c_{11}-\Sigma \Sigma^{H}\left(c_{12}+c_{21}\right)}{c_{11}^{2}}=0$$

where $\left\{ \begin{array}{l} \Delta ={\dot{s}}_{\theta }^{H}\left(\eta \right)R_{0}^{-1}x_{1}\\ \Sigma =s^{H}\left(\eta \right)R_{0}^{-1}x_{1}\\ c_{11}=s^{H}\left(\eta \right)R_{0}^{-1}s^{H}\left(\eta \right)\\ c_{12}=s^{H}\left(\eta \right)R_{0}^{-1}{\dot{s}}_{\theta }^{H}\left(\eta \right)\\ c_{21}={\dot{s}}_{\theta }^{H}\left(\eta \right)R_{0}^{-1}s\left(\eta \right) \end{array}\right.$

where ${\dot{s}}_{\theta }^{}\left(\eta \right)$ represents the first-order partial derivative of s(η) with respect to θ.

Simplifying (11), ${\dot{L}}_{\theta }\left(\eta \right)$ can be expressed as

(12) L˙θ(η)=Re(s˙θH(η)R0−1x1sH(η)R0−1x1)−Re(s˙θH(η)R0−1s(η)sH(η)R0−1s(η))=0

Rearranging (12), the equation can be written as

(13) Re(wθH(η)x1wH(η)x1)=Re(wθH(η)s(η)wH(η)s(η)) 

where $w_{\theta }^{}\left(\eta \right)=R_{0}^{-1}{\dot{s}}_{\theta }\left(\eta \right)$ and $w\left(\eta \right)=R_{0}^{-1}s\left(\eta \right)$ can be considered as the parameter-dependent adaptive sum and difference weight vectors.

Since the equations in (13) are complex nonlinear functions, the solution cannot be solved in analytical form. Observing (13), we can see that the complexity of (13) mainly results from the fact that the sum and difference weight vectors are functions of η. By searching the values of η, the weight vectors can obtain more matching results with the echo data, and thus the optimal solution is guaranteed. Although (13) can obtain the optimal solution of parameter estimation, the computational complexity resulted from fine grid-search is unacceptable. In practice, the performance improvement due to the variation of η only in one resolution is trivial. Thus, we can approximate the ML solution by setting the parameter-independent adaptive sum and difference weight vectors in (13), that is, $w_{\theta }^{}\left(\eta_{0}\right)=R_{0}^{-1}{\dot{s}}_{\theta }\left(\eta_{0}\right)$ and $w\left(\eta_{0}\right)=R_{0}^{-1}s\left(\eta_{0}\right)$, then (13) can be simplified as

(14) Re(wθH(η0)x1wH(η0)x1)=Re(wθH(η0)s(η)wH(η0)s(η)) 

Similarly, ${\dot{L}}_{v}\left(\eta \right)$ can be written as

(15) L˙v(η)=Re(s˙vH(η)R0−1x1sH(η)R0−1x1)−Re(s˙vH(η)R0−1s(η)sH(η)R0−1s(η))=0

Approximating (15) by using the parameter-independent adaptive sum and difference weight vectors, (15) can be approximated by

(16) Re(wvH(η0)x1wH(η0)x1)=Re(wvH(η0)s(η)wH(η0)s(η)) 

where $w_{v}^{}\left(\eta_{0}\right)=R_{0}^{-1}{\dot{s}}_{v}\left(\eta_{0}\right)$.

Meanwhile, ${\dot{L}}_{r}\left(\eta \right)$ can be written as

(17) L˙r(η)=Re(s˙rH(η)R0−1x1sH(η)R0−1x1)−Re(s˙rH(η)R0−1s(η)sH(η)R0−1s(η))=0

and (17) can be approximated by

(18) Re(wrH(η0)x1wH(η0)x1)=Re(wrH(η0)s(η)wH(η0)s(η)) 

where $w_{r}^{}\left(\eta_{0}\right)=R_{0}^{-1}{\dot{s}}_{r}\left(\eta_{0}\right)$.

Finally, the ML solution can be obtained by solving Equations (14), (16) and (18). Define Fi(η)=Re(wiH(η0)s(η)/(wH(η0)s(η))),i=θ,r,v as the response curve of target parameters and ηi=Re(wiH(η0)x1wH(η0)x1),i=θ,r,v as the difference–sum–ratio output. Then (14), (16) and (18) can be written as

(19) $$\left[\begin{array}{l} F_{\theta }\left(\eta \right)\\ F_{v}\left(\eta \right)\\ F_{r}\left(\eta \right) \end{array}\right]=\left[\begin{array}{l} \eta_{\theta }\\ \eta_{v}\\ \eta_{r} \end{array}\right]$$

To acquire the solution of (19), one-order Taylor approximation to $F_{i}\left(\eta \right)$ can be employed

(20) $$F_{i}\left(\eta \right)=p_{i}+\delta_{i\theta }\left(\theta -\theta_{0}\right)+\delta_{iv}\left(v-v_{0}\right)+\delta_{ir}\left(r-r_{0}\right),i=\theta ,r,v$$

where $p_{i}=F_{i}\left(\eta \right){|}_{\eta =\eta_{0}}$, $\delta_{i\theta }=\partial F_{i}\left(\eta \right)/\partial \theta {|}_{\eta =\eta_{0}}$, $\delta_{iv}=\partial F_{i}\left(\eta \right)/\partial v{|}_{\eta =\eta_{0}}$, $\delta_{ir}=\partial F_{i}\left(\eta \right)/\partial r{|}_{\eta =\eta_{0}}$, and ∂ means the partial derivative operator. After some manipulations, we have

{δiθ=Re((sH(η)R0−1s(η))(s¨iθH(η)R0−1s(η)+s˙iH(η)R0−1s˙θ(η))−(s˙iH(η)R0−1s(η))(s˙θH(η)R0−1s(η)+sH(η)R0−1s˙θ(η))(sH(η)R0−1s(η))2)δiv=Re((sH(η)R0−1s(η))(s¨ivH(η)R0−1s(η)+s˙iH(η)R0−1s˙v(η))−(s˙iH(η)R0−1s(η))(s˙vH(η)R0−1s(η)+sH(η)R0−1s˙v(η))(sH(η)R0−1s(η))2)δir=Re((sH(η)R0−1s(η))(s¨irH(η)R0−1s(η)+s˙iH(η)R0−1s˙r(η))−(s˙iH(η)R0−1s(η))(s˙rH(η)R0−1s(η)+sH(η)R0−1s˙r(η))(sH(η)R0−1s(η))2) and ${\ddot{s}}_{ij}^{H}\left(\eta \right)$ represents the second-order partial derivative of s(η) with respect to i and j.

Reformulating (20) into a matrix form yields

(21) $$\left[\begin{array}{l} F_{\theta }\left(\eta \right)\\ F_{v}\left(\eta \right)\\ F_{r}\left(\eta \right) \end{array}\right]=\left[\begin{array}{l} p_{\theta }\\ p_{v}\\ p_{r} \end{array}\right]+\left[\begin{array}{lll} \delta_{\theta \theta } & \delta_{\theta v} & \delta_{\theta r}\\ \delta_{v\theta } & \delta_{vv} & \delta_{vr}\\ \delta_{r\theta } & \delta_{rv} & \delta_{rr} \end{array}\right]\cdot \left[\begin{array}{l} \theta -\theta_{0}\\ v-v_{0}\\ r-r_{0} \end{array}\right]$$

Substituting (21) into (19) yields

(22) $$\left[\begin{array}{l} \theta \\ v\\ r \end{array}\right]=\left[\begin{array}{l} \theta_{0}\\ v_{0}\\ r_{0} \end{array}\right]+{\left[\begin{array}{lll} \delta_{\theta \theta } & \delta_{\theta v} & \delta_{\theta r}\\ \delta_{v\theta } & \delta_{vv} & \delta_{vr}\\ \delta_{r\theta } & \delta_{rv} & \delta_{rr} \end{array}\right]}^{-1}\cdot \left(\left[\begin{array}{l} \eta_{\theta }\\ \eta_{v}\\ \eta_{r} \end{array}\right]-\left[\begin{array}{l} p_{\theta }\\ p_{v}\\ p_{r} \end{array}\right]\right)$$

**Figure 1:**
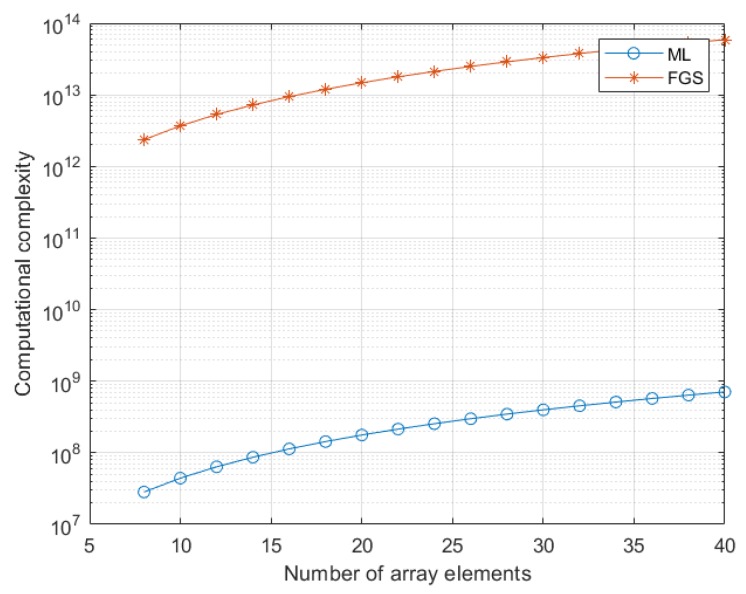
Computational complexities of the multi-dimensional maximum likelihood (ML) and fine grid-search (FGS) methods when the number of array elements varies from 8 to 40.

To improve the accuracy of Taylor approximation, the iterative technique can be employed, which uses the estimated target parameters instead of the assumed target parameters, i.e., $\left[\theta_{0},v_{0},r_{0}]=[\hat{\theta },\hat{v},\hat{r}\right]$. It is shown from (22) that target parameter estimation can be realized by a simple expression, where the computational complexity is $O\left(12{\left(NM\right)}^{2}+12NM+3^{3}\right)$ complex multiplications. Due to the fact that the computational complexity of complex addition is less than that of complex multiplication, complex additions are not considered here. In contrast, the conventional grid-search technique has the computational complexity of $O\left(\left({\left(NM\right)}^{2}+2NM\right)J^{3}\right)$ complex multiplications, where J is the number of grids in one dimension. To guarantee the accuracy, the number of grids is usually larger than 100. To demonstrate the advantage of the computational complexity, the computational complexities of the presented method and the grid-search method as a function of the number of array elements are plotted in Figure 1, where the number of grids is 100, and the number of pulses is 192. It is observed that the presented method owns lower computational complexity than the fine grid-search method.

## 3. Numerical Experiments

In this section, the effectiveness of the proposed method is verified by simulation experiments. The radar parameters are designed as follows: a uniform linear array with 8 elements is employed, the transmitter transmits a multi-cycle inter-pulse stepped frequency signal by 192 pulses, each group has 16 pulses, and the carrier frequency hops from 2 to 2.016 GHz with the frequency increment of 1 MHz, the sampling frequency is 16 MHz, and the pulse repetition frequency is 4000 Hz.

### 3.1. Deceptive Interference Suppression and Target Parameter Estimation for Frequency Agile Radar

A frequency agile radar is designed based on the above parameters, and the synthesized bandwidth is increased by 16 times. Therefore, under the same parameters, the range resolution of the frequency agile radar is 16 times that of the fixed frequency radar. By using the bandwidth synthesis function of FAR [13], 16 fine range gates in each coarse range unit can be obtained. Thus, distance information can be estimated accurately.

**Figure 2:**
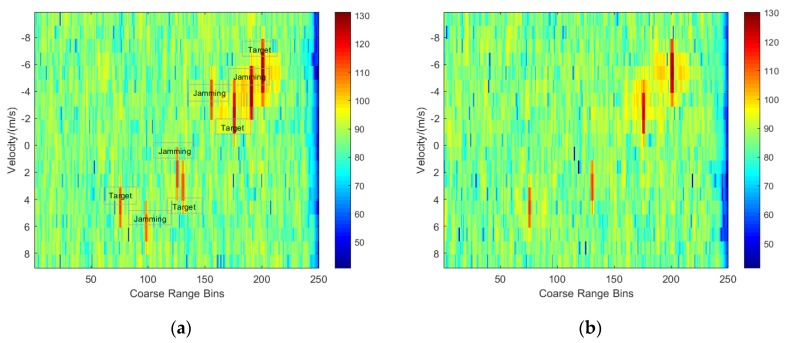
Echo plane after matched filtering. (**a**) Frequency non-agile case; (**b**) Frequency agile case

To analyze the performance of suppressing deceptive jamming, the experiment randomly simulates multiple targets and multiple deceptive jamming located in various coarse range bins, velocities and distances. Figure 2 gives the echo plane after matched filtering for the frequency non-agile case and the frequency agile case, where multiple target signals and deceptive jamming signals with random parameters are simulated, where the targets are located in the range bins of 76, 131, 176 and 201, respectively, and the jamming signals are located in the range bins of 99, 126, 156 and 191, respectively. It is observed that the target signals and the deceptive jamming signals are all preserved for the frequency non-agile case in Figure 2a, whereas only the target signals are well preserved for the frequency agile case in Figure 2b.

In order to see the performance of suppressing deceptive jamming and range resolution enhancement resulted from frequency agility, three target signals are added in the echo data, where the two targets are set at the 71st coarse range bin and another one is located in the coarse range bin of 114. Meanwhile, four deceptive jamming signals are added, where two deceptive jamming signals are set at the 110th coarse range bin and others are located in the coarse range bin of 149. Additionally, the signals at the same range bin have different speeds and angles.

**Figure 3:**
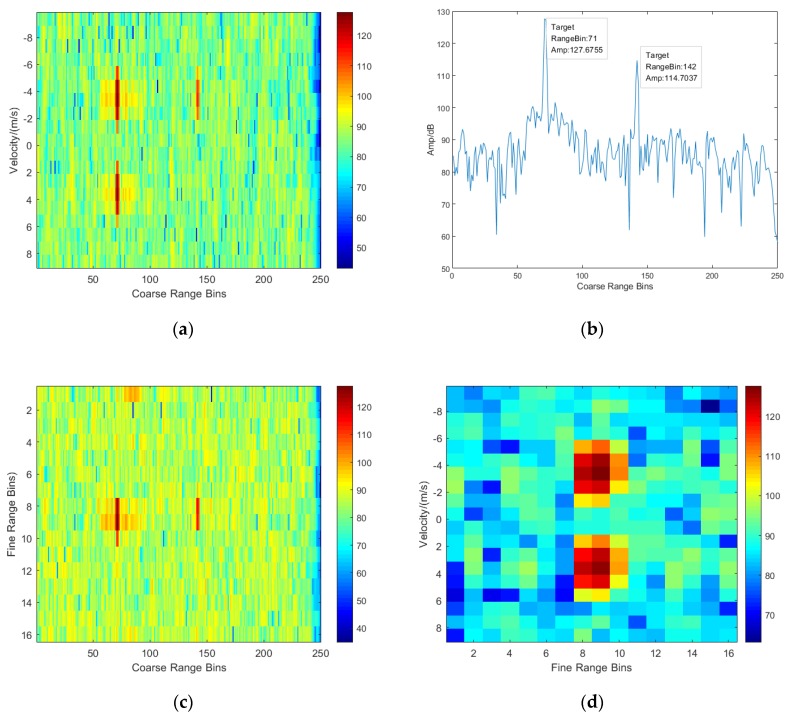
Echo plane after matched filtering for frequency-agile case. (**a**) Coarse range–velocity plane; (**b**) Range plot at the velocity of −3.5 m/s; (**c**) Coarse range–fine range plane; (**d**) Fine range–velocity plane.

**Figure 4:**
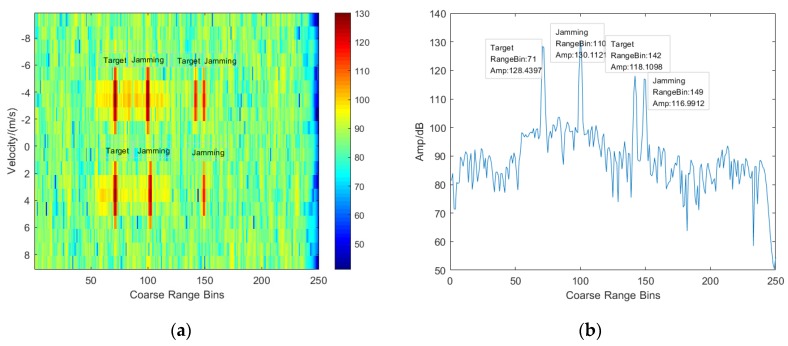
Echo plane after matched filtering for frequency non-agile case. (**a**) Coarse range–velocity plane; (**b**) Range plot at the velocity of −3.5 m/s.

The matched filtering results for the frequency agile case and the frequency non-agile case are given in Figure 3 and Figure 4. Comparing the plane of coarse range bin and velocity given in Figure 3a and Figure 4a, it is clearly observed that the target signal is preserved, and the deceptive jamming is suppressed due to the agile carrier frequency. Figure 3b and Figure 4b are the range cuts of Figure 3a and Figure 4a at the velocity of −3.5 m/s, which show a perfect performance of deceptive jamming suppression for the frequency agile case. Furthermore, a plane of coarse range bin and fine range bin given in Figure 3c indicates that more accurate distance information can be obtained since there is a fine resolving within coarse range bin. In addition, Figure 3d shows the estimation of target speed and accurate distance.

### 3.2. Estimation of Target Parameters in Various Conditions

In this section, the simulation results in various conditions are carried out to validate the effectiveness of the proposed algorithm. Experiments of the fine grid-search method [21] and the adaptive monopulse method [12] are also performed to prove the superiority of the proposed method. All results are obtained by running a Monte Carlo simulation, where the number of Monte Carlo experiments is 500. For simplicity, the normalized root mean square error (NRMSE) is employed here, which is defined as ${\bar{\sigma }}_{i}=\sigma_{i}/\rho_{i},i=[\theta ,v,r]$, where $\sigma_{i}$ is the classical RMSE, and $\rho_{i}$ is the resolution. In this simulation, the resolution of $\rho_{\theta }$ is 14.32°, the resolution of $\rho_{v}$ is 1.56 m/s, and the resolution of $\rho_{r}$ is 9.37 m. The target and deceptive jamming are located at the range bins from 1500 to 3000 randomly, the velocity of them are from −6 to 6 m/s.

*A.* *NRMSE in Signal Noise Ratio (SNR) Cases*

**Figure 5:**
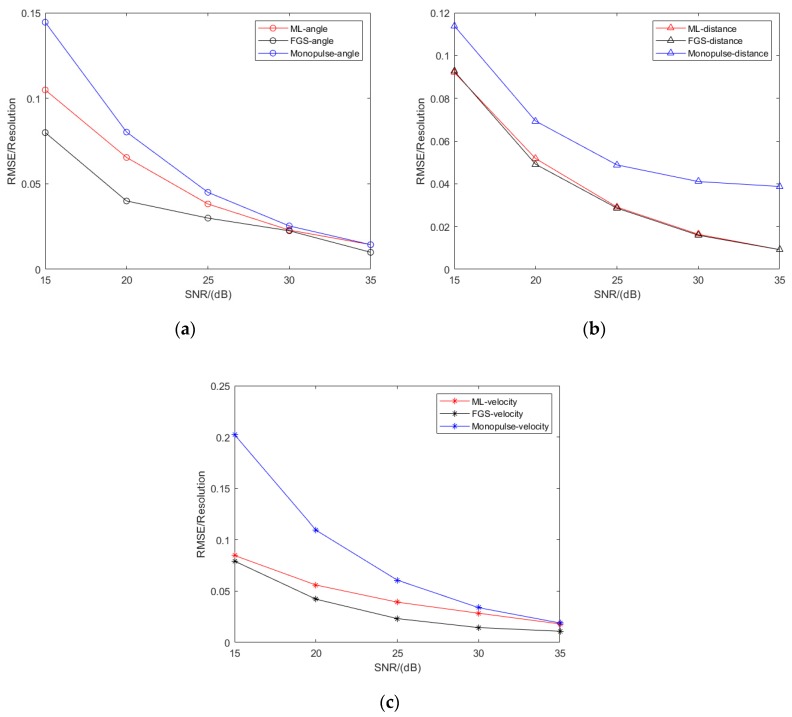
Statistical performance results in SNR cases. (**a**) Angle estimation results; (**b**) Range estimation results; (**c**) Velocity estimation results.

In Figure 5, the NRMSEs as a function of SNR are given, where the SNR varies from 15 to 35 dB, the line of sight is 113° and the angle of the target is 113.5°. Parameter estimates of angle, range and velocity by three methods are also incorporated. It is shown that RMSEs of target parameters decrease with the SNR increasing, and the RMSEs of our presented method approach those of the fine grid-search method in most of SNR cases. Meanwhile, our presented method shows better estimated accuracy than the adaptive monopulse method.

*B.* *NRMSE in the Number of Barrage Noise Jamming Cases*

**Figure 6:**
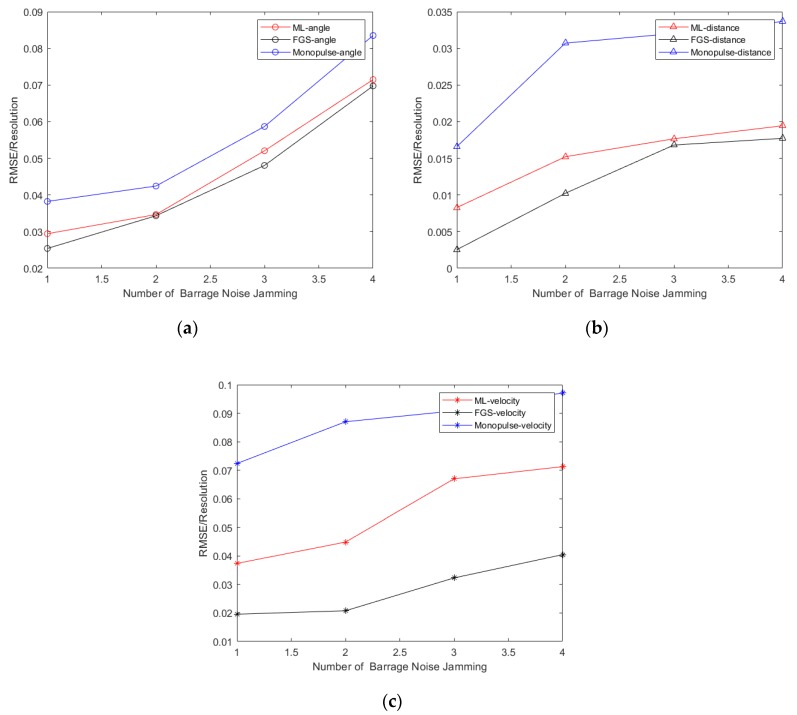
Statistical performance results with the number of barrage noise jamming increasing. (**a**) Angle estimation results; (**b**) Range estimation results; (**c**) Velocity estimation results.

In Figure 6, the NRMSEs of angle, range and velocity as a function of the number of barrage noise jamming are given for the three methods, the line of sight is 113° and the angle of the target is 113.5°. It should be noted that as the number of barrage noise jamming varies from one to four, the RMSEs of target parameters will increase, which means the estimated accuracy is decreasing. Certainly, the target parameters estimated by the fine grid-search method is the closest to the actual values, followed by the ML method, and the adaptive monopulse method.

*C.* *NRMSE in the Angle Deviation from the Line of Sight Cases*

**Figure 7:**
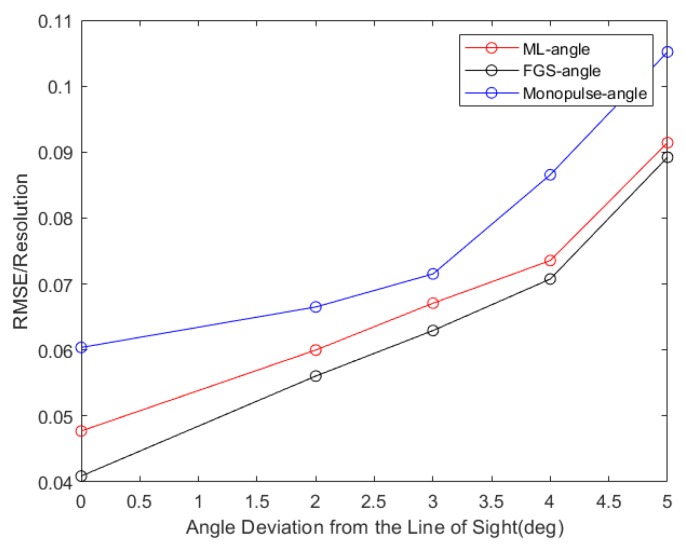
Statistical performance results versus angle deviation from the line of sight.

In Figure 7, the NRMSEs of the target angle versus angle deviation from the line of sight are depicted using three methods. The line of sight is 113°, where the deviation range between the target and the look direction is from 0° to 5°. It is observed that the RMSEs increase and the estimated performance decreases as the target angle deviates from the look direction. As shown in Figure 6, it can be clearly seen that the target parameters are finely estimated by the fine grid-search method and roughly estimated by the adaptive monopulse method, the ML method may achieve a good balance between them.

*D.* *NRMSE in the Line of Sight Cases*

**Figure 8:**
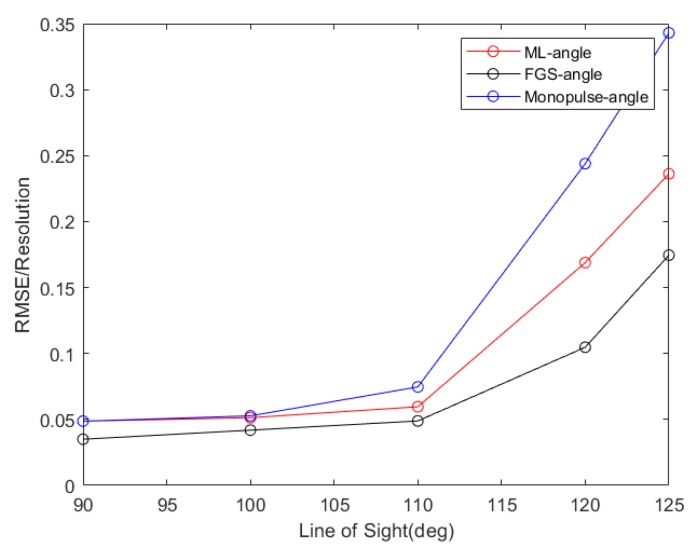
Statistical performance results with the line of sight increasing.

In Figure 8, the NRMSEs as a function of the line of sight are given, where the look direction varies from 90° to 125° and the deviation between the target and the look direction is 0.5°, it is shown that as the look direction deviates from the normal direction, the RMSEs increase, and the parameter estimation performance decreases. Although the performance of the presented method is worse than that of the FGS method, it has a better performance compared to the adaptive monopulse method.

## 4. Conclusions

In this communication, fast approximated ML estimation for FAR under the sophisticated jamming environment including the deceptive jamming and the barrage noise jamming is considered. The presented method suppresses the barrage jamming by adaptive beamforming and the deceptive jamming by frequency agility. To reduce the computational complexity, we estimate the target parameters by a low-order approximation of the multi-dimensional ML function. Thus, the proposed method may achieve a good balance between the computational cost and the target parameter estimation performance. Numerical examples are given to demonstrate the effectiveness of the presented method.
